# Thirty-day readmission rates, trends and its impact on liver transplantation recipients: a national analysis

**DOI:** 10.1038/s41598-020-76396-5

**Published:** 2020-11-06

**Authors:** Khalid Mumtaz, Jannel Lee-Allen, Kyle Porter, Sean Kelly, James Hanje, Lanla F. Conteh, Anthony J. Michaels, Ashraf El-Hinnawi, Ken Washburn, Sylvester M. Black, Marwan S. Abougergi

**Affiliations:** 1 Division of Gastroenterology, Hepatology and Nutrition, The Ohio State University Wexner Medical Center, 395 West 12th Ave., 3rd Floor, Columbus, OH 43210 USA; 2 Department of Internal Medicine, The Ohio State University Wexner Medical Center, Columbus, OH USA; 3 Center for Biostatistics, Department of Biomedical Informatics, The Ohio State University, Columbus, OH USA; 4 Division of Transplantation, Department of Surgery, The Ohio State University Wexner Medical Center, Columbus, USA; 5 Division of Gastroenterology, Department of Internal Medicine, University of South Carolina, Columbia, SC USA; 6 Catalyst Medical Consulting, Simpsonville, USA

**Keywords:** Hepatic artery, Bile ducts, Liver cirrhosis

## Abstract

Reduction of early hospital readmissions is a declared goal in the United States economic and quality improvement agenda. A retrospective study was performed using the Nationwide Readmissions Database from 2010 to 2014. Our primary aim was to study the rate of early readmissions and its predictors in liver transplant recipients (LTRs). Our secondary aims were to determine the trends of LT, reasons for readmission, costs and predictors of calendar year mortality. Multivariable logistic regression and Cox proportional hazards models were utilized. The 30-day readmission rate was 30.6% among a total of 25,054 LTRs. Trends of LT were observed to be increased in patients > 65 years (11.7–17.8%, *p* < 0.001) and decreased in 40–64 years (78.0–73.5%, *p* = 0.001) during study period. The majority of 30-day readmissions were due to post transplant complications, with packed red blood cell transfusions being the most common intervention during readmission. Medicaid or Medicare insurance, surgery at low and medium volume centers, infections, hemodialysis, liver biopsy, and length of stay > 10 days were the predictors of 30-day readmission. Moreover, number of early readmission, age > 64 years, non-alcoholic cirrhosis, and length of stay > 10 days were significant predictor of calendar year mortality in LTRs. Approximately one third of patients require early admission after LT. Early readmission not only increases burden on healthcare, but is also associated with calendar year mortality. Strategies should be implemented to reduce readmission in patients with high risk of readmission identified in our study.

## Introduction

With the recent advances in surgical technique and medical care, liver transplantation (LT) is now a successful surgery, with resulting improvement in median patient life expectancy from 2 years without LT to approximately 10 years with LT^1^. However, patient evaluation for LT, the listing process and waiting list as well as the surgery itself is resource intensive and not without challenges. Studies have reported the mortality rate during the initial hospitalization to be 5% -10% with an associated increased total hospitalization costs^2–7^. After discharge, early hospital readmission has a significant contribution to mortality and resource utilization. Several single center studies and one study using the UNOS database have investigated the predictors, healthcare utilization and outcomes of post-LT early readmission^2–6^. The reported readmission rates in these studies have ranged from 35 to 50%. Recipient old age, altered mental status, high Model for End-Stage Liver Disease (MELD) score, need for mechanical ventilation, and multiple co-morbidities were identified as best predictors of post LT early readmission^3–5^. To date, the study that has included the largest number of patients is that using the UNOS database linked to University Health System Consortium from 2007 to 2011 by Wilson et al.^6^. In the study, the authors reported a 30-day readmission rate after LT of 38%. The independent predictors of readmission were a high MELD score, Diabetes Mellitus at time of LT, renal dialysis dependence at time of LT, a high donor risk index allograft and discharge to a rehabilitation facility.

Reduction of hospital readmissions is a declared goal in the United States economic and quality improvement agenda. The Affordable Care Act (ACA) mandated the public reporting of 30-day hospital readmission rates for several medical and surgical conditions. This step is a necessary precursor for the implementation of penalties (up to 3% reduction in reimbursements) to hospitals with elevated readmission rates^8^. Initially, these penalties applied for certain medical conditions including heart failure, pneumonia and acute myocardial infarction as well as some elective surgeries including total hip and/or knee arthroplasty. More recently, coronary artery bypass grafting has been added to the list^8^. While LT is not currently a part of the Hospital Readmission Reduction Program (HRRP), there are often indirect financial consequences to hospitals, as many managed care plans offer a fixed reimbursement for this procedure^3^. Most importantly, 30-day readmission is associated with increased morbidity and mortality for patients^3–6^. The last reported LT national readmission rate and its predictors was based on 2007–2011 data. This data is based on a very comprehensive set of databases that captured the majority, yet not all, LT recipients. Therefore, we set out to provide updated statistics for early LT readmission rates and its predictors using recent releases of the largest publicly available readmission database in the United States. We also aimed to study the reasons for readmission as well as the impact of early readmission on calendar year mortality and healthcare resource utilization.

## Methods

### Database information

We utilized the 2010–2014 Nationwide Readmissions Database (NRD) to study 30-day readmissions among LT recipients. The NRD is a part of the Healthcare Cost and Utilization Project (HCUP), a government initiated group of databases. It is the nation’s largest database of all-payer, encounter-level hospital care data and is developed through a Federal-State-Industry partnership and sponsored by the Agency for Healthcare Research and Quality (AHRQ)^9^. The NRD is the HCUP database that focuses exclusively on hospital readmissions via verified, de-identified, but linked patient data from HCUP state inpatient databases. It is designed as a stratified probability sample to be representative of all nonfederal acute care inpatient hospitalizations nationwide.

Briefly, hospitals are stratified according to ownership/control, number of beds, teaching status, urban/rural location, and geographic region. A 20% probability sample of all hospitals within each stratum is then collected. Those hospital discharges are recorded, and information about patients’ demographics, principal and secondary diagnoses, vital status at discharge, readmission and resource use including length of stay, procedures performed, and total hospitalization costs and charges are entered into the NRD. Each discharge is then weighted (weight = total number of discharges from all acute care hospitals in the United States divided by the number of discharges included in the 20% sample) to make the NRD nationally representative^8^.

The NRD contains both patient- and hospital-level information. Up to 25 discharge diagnoses and 15 procedures are collected for each patient using the International Classification of Diseases, Ninth Revision, Clinical Modification (ICD9-CM). The NRD utilizes the verified patient linkage numbers in the state inpatient database (SID) to track each patient with any type of insurance or no insurance across hospitals within a state. From 2010 to 2014, the NRD collected data from 13.9 to 14.9 million discharges each year from 1808 to 2048 hospitals across 18 to 22 geographically dispersed states, accounting for over 49% of both the total United States resident population and hospitalizations.

The Ohio State Data and Specimen Policy and Human Subjects Research policy does not require Institutional Review Board (IRB) approval for population based public data set. Per 45 Code of Federal Regulations (CFR 46.101), research using certain publicly available data sets does not involve “human subjects”. The data contained within these specific data sets are neither identifiable nor private and thus do not meet the federal definition of “human subject” as defined in 45 CFR 46.102. Therefore these research projects do not need to be reviewed and approved by the IRB".

### Study population

**Figure 1:**
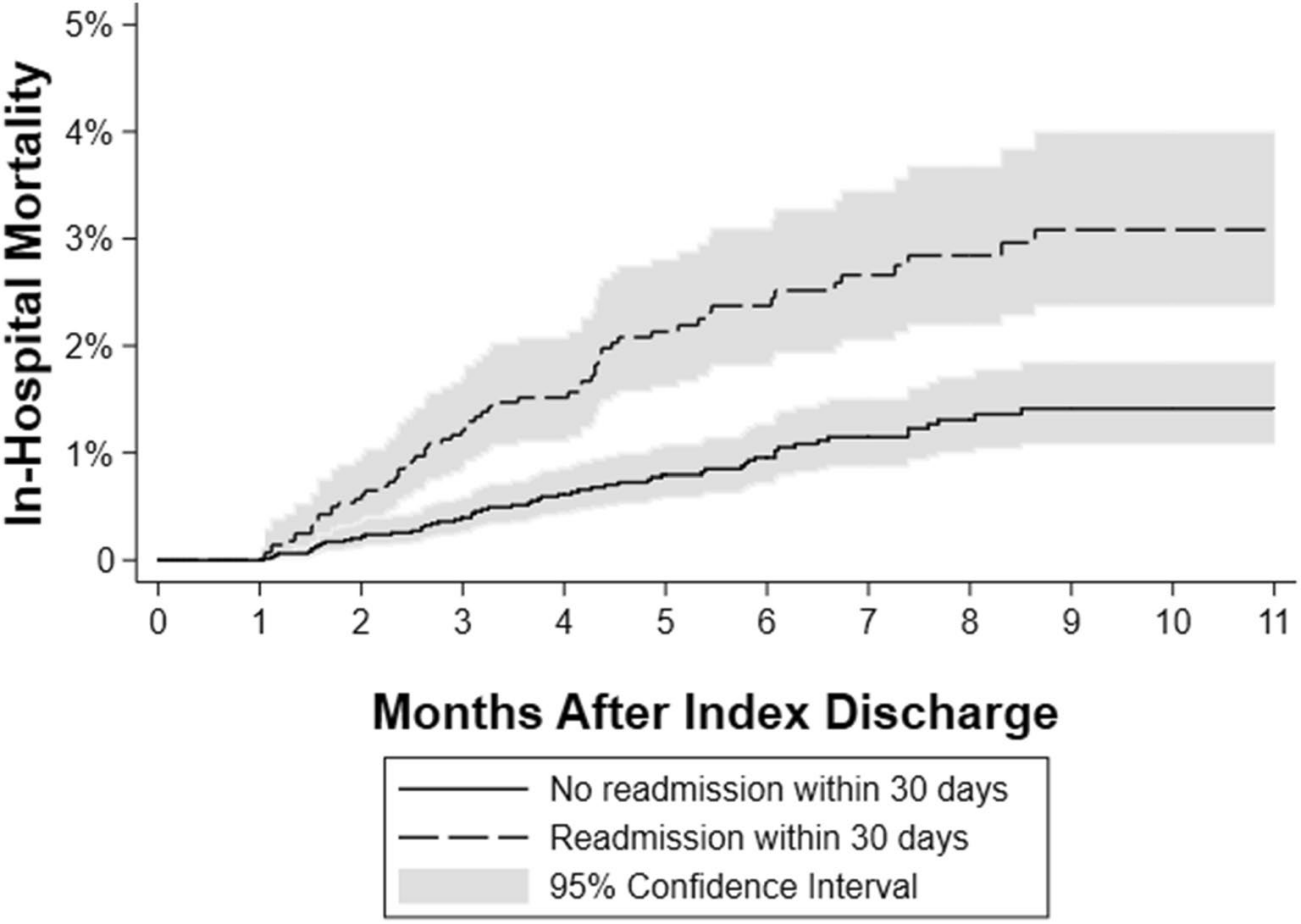
Patient selection.

Patients who underwent deceased donor liver transplantation between January 2010 and November 2014 were included in the study. We excluded (1) patients less than 18 years of age (n = 3777), (2) patients receiving multi-organ transplants (n = 3620), (3) re-liver transplantation (n = 0), (4) those discharged from the hospital during the month of December because of unavailability of a full 30 days of follow-up period to ascertain the occurrence of readmission (n = 3114), and (5) patients who died during the index hospitalization (n = 1431; Fig. 1). There is no separate ICD code for re- liver transplantation (re-LT). However, we defined the index admission for each patient as the first instance of an eligible LT. A subsequent LT would not be included as an index admission.

All the ICD-9 CM procedure and diagnosis codes used in this study are presented in Supplementary Table 1. The etiology of patients’ liver disease was classified as nonalcoholic unless alcohol dependence was listed as a diagnosis. Patients were considered to have liver cirrhosis only if a diagnosis of liver cirrhosis was present during the index admission. The Baveno IV criteria were used to identify patients with decompensated liver cirrhosis^10^. Baveno stages I through IV are defined as: I- no varices or ascites; II- varices without ascites; III- ascites with or without varices; and IV- bleeding varices with or without ascites, respectively^10^.

**Table 1:** Patient and hospital demographics in liver transplant recipients.

Variable	All patients (n = 25,054)	Readmission within 30 days of index discharge (n = 7675; 30.6%)	No readmission within 30 days of index discharge (n = 17,379; 69.4%)	Effect size	*p* value
Age group				0.04	0.28
18–39	2109 (8.4%)	702 (9.1%)	1407 (8.1%)		
40–64	19,199 (76.6%)	5866 (76.4%)	13,333 (76.7%)		
> 64	3746 (15.0%)	1107 (14.4%)	2639 (15.2%)		
Gender				0.02	0.39
Male	16,826 (67.2%)	5103 (66.5%)	11,724 (67.5%)		
Female	8228 (32.8%)	2573 (33.5%)	5655 (32.5%)		
Primary payer				0.13	0.01
Medicare	6841 (27.3%)	2245 (29.3%)	4596 (26.4%)		
Medicaid	3666 (14.6%)	1280 (16.7%)	2386 (13.7%)		
Private Insurance	12,942 (51.7%)	3716 (48.4%)	9226 (53.1%)		
Other/Unknown	1605 (6.4%)	434 (5.7%)	1171 (6.7%)		
Income Quartile				0.03	0.98
1st Quartile	6092 (24.4%)	1868 (24.3%)	4224 (24.4%)		
2nd Quartile	6417 (25.6%)	1940 (25.3%)	4477 (25.8%)		
3rd Quartile	6332 (25.3%)	1974 (25.7%)	4358 (25.1%)		
4th Quartile	5507 (22.0%)	1690 (22.0%)	3817 (22.0%)		
Missing	669 (2.7%)	202 (2.6%)	467 (2.7%)		
Hospital Liver Transplant Volume				0.12	< 0.001
Low (1–48/year)	6063 (24.2%)	2070 (27.0%)	3993 (23.0%)		
Medium (49–77/year)	5200 (20.8%)	1674 (21.8%)	3526 (20.3%)		
High (78+/year)	13,790 (55.0%)	3931 (51.2%)	9859 (56.7%)		
Cirrhosis type				0.07	0.01
Compensated cirrhosis or no cirrhosis code	11,595 (46.3%)	3364 (43.8%)	8231 (47.4%)		
Decompensated cirrhosis	13,459 (53.7%)	4312 (56.2%)	9147 (52.6%)		
Cirrhosis etiology				0.01	0.82
Nonalcoholic	18,715 (74.7%)	5746 (74.9%)	12,968 (74.6%)		
Alcoholic	6340 (25.3%)	1929 (25.1%)	4410 (25.4%)		
Ascites	12,072 (48.2%)	3919 (51.1%)	8153 (46.9%)	0.08	0.001
HCC	8373 (33.4%)	2345 (30.6%)	6028 (34.7%)	0.09	0.01
Diabetes Mellitus Type 2	6406 (25.6%)	1950 (25.4%)	4456 (25.6%)	0.01	0.84
Hepatic artery thrombosis	3806 (15.2%)	1288 (16.8%)	2518 (14.5%)	0.03	0.20
Biliary Complications	2020 (8.1%)	684 (8.9%)	1336 (7.7%)	0.06	0.02
Shock, including sepsis/septic	8207 (32.8%)	2855 (37.2%)	5353 (30.8%)	0.04	0.07
Infection, not including Sepsis	2109 (8.4%)	702 (9.1%)	1407 (8.1%)	0.14	< 0.001

### Patient and hospital characteristics

Data collected for each patient and directly provided in NRD included age, sex, insurance type, income based on the zip code, length of stay (LOS), discharge disposition. The etiology of liver cirrhosis was divided into alcoholic and non-alcoholic (viral, non-alcoholic fatty liver disease, autoimmune, primary biliary cirrhosis, primary sclerosing cholangitis or cryptogenic); covariates of interest included hyponatremia, biliary complications (anastomotic strictures, bile leak), ascites, hepatocellular carcinoma (HCC), Diabetes Mellitus, sepsis/infections/clostridium difficile infection (CDI), hepatic artery thrombosis and requirement of vasopressors.

Surgical interventions and procedures, including mechanical ventilation, laparotomy, endoscopic retrograde cholangio-pancreaticography (ERCP), Gastrointestinal (GI) endoscopy, percutaneous biliary cholangiography, liver biopsy, percutaneous abdominal drainage, blood transfusion and hemodialysis were identified using ICD-9 CM codes (Supplementary Table 1). Comorbidities were assessed using the Elixhauser comorbidity index and stratified into < 3 or ≥ 3 points (modified to exclude liver disease)^11,12^. The Elixhauser comorbidity index uses ICD diagnosis codes to stratify comorbidities to predict hospital resource use and in-hospital mortality^11,12^. It has been validated for use with administrative hospital databases^12^.

Annual LT volume for each hospital was stratified as low (1–48/year), medium (49–77/year) and high (78+/year)^13,14^. Finally, the length of stay (LOS), as well as total admission charges and cost were collected. Total hospitalization charges for index admission are directly provided in NRD. To obtain the total hospitalization costs, total hospitalization charges were multiplied by the provided corresponding cost-to-charge ratio. HCUP obtains the cost-to-charge ratios from the hospital accounting reports collected by the Centers for Medicare and Medicaid Services. (US Agency for Healthcare Research and quality cost-to charge ratio files. https://www.hcup-us.ahrq.gov/db/state/costtocharge.jsp. Accessed February 13, 2019). Hospital yearly volume was calculated by adding all the admissions for LT at each hospital during each study year. Hospital volume was divided into tertiles for all analyses.

### Outcomes

The primary outcome was 30-day readmission rate and its predictors in liver transplant recipients (LTRs). 30-day readmission was defined as any hospital admission following the index discharge for any reason^3^. Time to readmission was calculated from the discharge date of the index admission. Readmissions occurring in the same states and same calendar year are identifiable in NRD. The secondary outcomes were: (a) Trends of 30-day readmission, in-hospital mortality and total hospitalization costs after LTs from 2010 to 2014; (b) most common reasons for readmission based only on the first readmission within 30 days, (c) total hospitalization costs associated with calendar-year readmission and (d) independent predictors of calendar-year mortality.

### Missing data

Admissions with missing total hospitalization charges (n = 296) were excluded from the cost analysis. The only other two variables in which data was missing were primary payer and patient’s zip code median income quartile. For the primary payer variable, the missing values (n = 339; 1.3%) were categorized with the “Other/Unknown” category. For the patient’s zip code median income quartile variable, the missing values (n = 669; 1.9%) were categorized as a separate “Missing” category.

### Statistical analysis

Statistical analyses were performed using SAS 9.4. (SAS Institute, Cary, NC). The NRD is based on a complex sampling design that includes stratification, clustering, and weighting. This software facilitates analysis to produce nationally representative unbiased results, variance estimates, and *p* values. Weighting of patient-level observations was implemented to all analyses to obtain estimates for the entire population in the United States of hospitalized patients for LT. Temporal trends were tested by linear regression for number of liver transplants (yearly estimates with inverse variance weighting) and total hospital costs (log-transformed) and by logistic regression for rates of 30 day-readmission, calendar year mortality, and interventions. Total hospitalization costs were adjusted for inflation over the study period using the personal health care (hospital care) price indices from the Medical Expenditure Panel Survey (MEPS) and presented in 2014 dollars. Independent predictors of 30-day readmissions were identified by univariable and multivariable logistic regression. Independent predictors of calendar year mortality were identified using a multivariable Cox proportional hazards model. Since the aim of the analysis was to evaluate the association between readmission within 30 days and mortality, all patients who died within 30 days of index discharge were considered censoring events and censored at the time of mortality. Patients who survived until the end of the calendar year were censored at that time point. For both multivariable models, backward selection was utilized for variable selection, using the criteria of *p* < 0.05. Model assumptions were checked for each model: dependent variable structure and multi-collinearity for all, normally distributed independent errors for linear regression, and proportional hazards for Cox models (by assessment of Schoenfeld residuals and Kaplan–Meier curves). Multivariable model performance was summarized by the c-index (logistic regression) and Harrell’s concordance statistic (Cox proportional hazards). In both models, the 95% confidence interval for the performance statistic was estimated by non-parametric bootstrap resampling. One thousand samples with replacement of size 25,054 were selected, the selected model fit, and the performance measure calculated (c-index or Harrell’s concordance statistic). From the empirical distribution of the resampled performance statistics, the 0.025 and 0.975 percentiles were identified as the 95% confidence limits^15^. In the logistic regression and Cox proportional hazards models, results of omnibus tests are reported for categorical predictors with more than two levels; each omnibus test is the multiple degree of freedom test of the null hypothesis that the odds or hazard rate is equal for all levels of the variable. In other analyses, proportions were compared using chi-square tests and continuous variables were compared using the Student t-test. Effect sizes were reported in summary tables as standardized mean differences using Cohen’s d for continuous variables and analogous calculations for categorical variables^16^. For variables summarized as median [IQR], the standardized mean differences were calculated from log-transformed data. All statistical tests were evaluated at a type I error rate of α = 0.05.

## Results

### Patient and hospital characteristics

A total of 25,054 patients who underwent liver transplantation between 2010 and 2014 were included in the study. Patient demographics, hospital characteristics and interventions during the index admission are summarized in Tables 1 and 2. Eight-five percent (n = 21,308) of the patients who underwent LT were under the age of 65 years. Approximately 42% (n = 10,507) of the patients were insured by Medicare or Medicaid. The patients were roughly equally distributed among the four income quartiles. Over half of the index admissions (n = 13,790) for LT were in high volume centers. The majority of patients underwent LT for non-alcoholic liver disease (75%; n = 18,715); ascites and HCC were reported for 48.2% (n = 12,072) and 33.4% (n = 8373) of patients, respectively. The median total hospitalization costs for LT index admission was $91,340 (Interquartile range (IQR) $67,480–$138,398) and the median length of stay (LOS) was 10.6 days (IQR 6.8–22.5). Notably, the LOS was greater than 10 days for 53.0% (n = 13,282) of patients (Table 2).

**Table 2:** Intervention and outcomes by 30-day readmission status.

Variable	All patients (n = 25,054)	Readmission within 30 days of index discharge (n = 7675; 30.6%)	No Readmission within 30 days of index discharge (n = 17,379; 69.4%)	Effect size	*p* value
Infusion of intravenous vasopressor	843 (3.4%)	272 (3.6%)	571 (3.3%)	0.01	0.67
Acute respiratory failure w/Mechanical ventilation	2544 (10.2%)	812 (10.6%)	1732 (10.0%)	0.02	0.88
Acute Kidney Injury w/Hemodialysis	2924 (11.7%)	1112 (14.5%)	1813 (10.4%)	0.12	< 0.001
Index admission LOS, median (IQR)	10.6 (6.8–22.5)	12.5 (7.4–25.1)	10.0 (6.7–21.2)	0.11	< 0.001
Index admission LOS > 10 days	13,282 (53.0%)	4560 (59.4%)	8722 (50.2%)	0.19	< 0.001
Total index admission cost ($), median (IQR)	91,340 (67,480–138,398)^a^	98,067 (71,531–143,466)^b^	84,672 (63,456–129,852)^c^	0.09	< 0.001

^a^n = 24,758 non-missing (98.8%).

^b^n = 7552 non-missing (98.4%).

^c^n = 17,206 non-missing (99.0%).

### Time trends of 30-day readmission rates and other outcomes

A total of 7675 (30.6%) of LT recipients were readmitted within 30 days; of these 1616 (21%) were readmitted more than once within 30 days. The median number of days from index discharge to first readmission was 8.1 days (IQR 3.6–15.6). A total of 6059 (24.2%) patients had 1 readmission within 30 days, 1431 (5.7%) had 2 readmissions within 30 days, 172 (0.7%) had 3 readmissions within 30 days, and 14 (0.1%) had 4 readmissions within 30 days.

**Figure 2:**
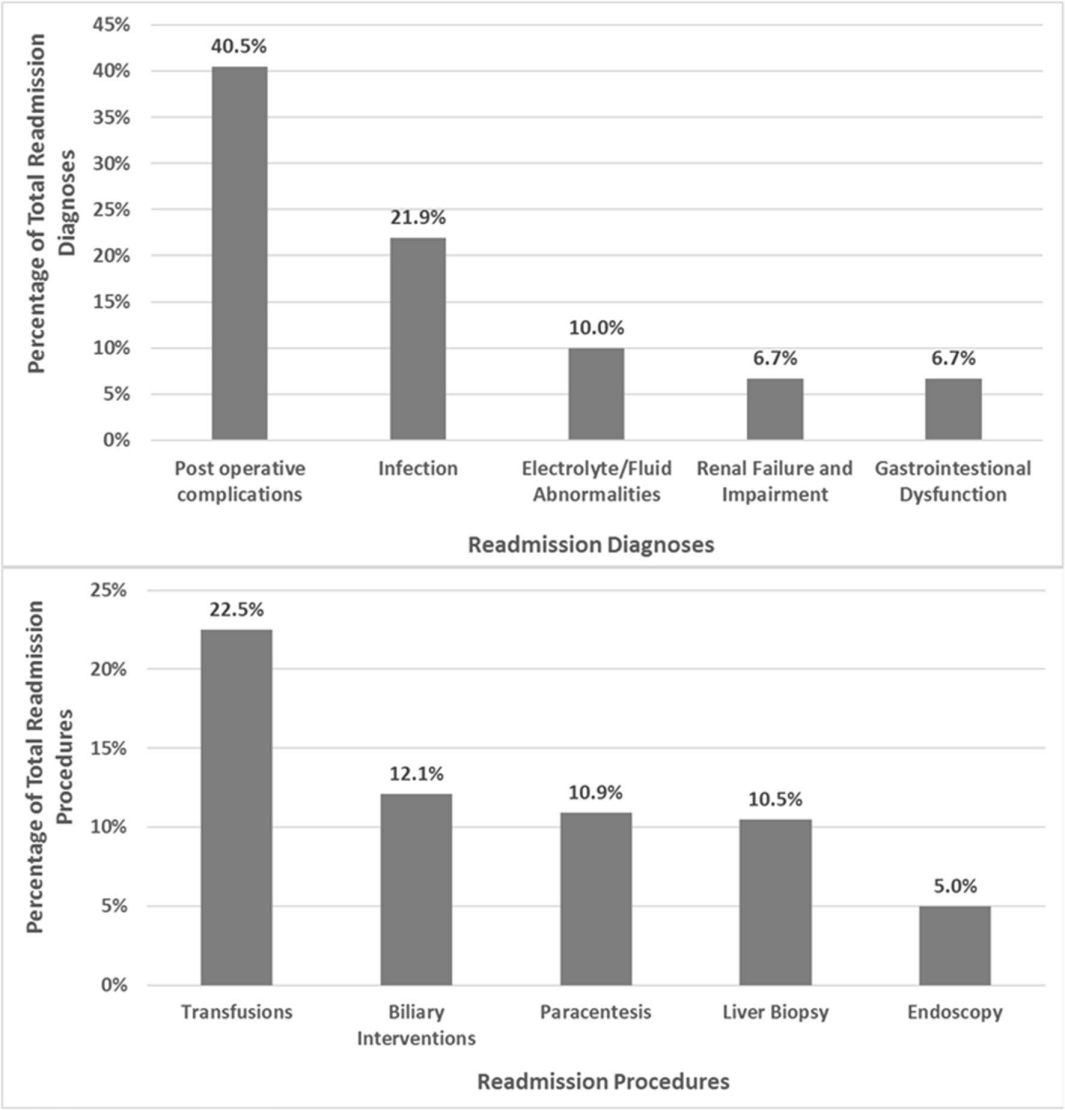
Trends of calendar year Mortality and its Predictors, 2010–2014.

Details of 30-day readmission as well as the other outcomes trends over time are presented in Fig. 2. The overall calendar year in-hospital mortality was 1.7%, and fluctuated between 1.4% and 2.0%. From 2010 to 2014, there was no statistically significant change in 30-day readmission rate (31.8–28.4%, *p* = 0.15). However, the percentage of liver transplant surgeries performed among patients older than 65 years increased (11.7–17.8%, *p* < 0.001) while it decreased among patients between 40 to 64 years of age (78.0–73.5%, *p* = 0.001). During the same time period, the inflation-adjusted total hospitalization costs increased by 14%, from $105,206 in 2010 to $119,967 in 2014. The same trend was seen for inflation adjusted calendar-year hospitalization costs. Along the same lines, the percentage of patients requiring renal hemodialysis increased from 12.1% in 2010 to 16.5% in 2014, (*p* = 0.04).

### Most common reasons for readmission

**Figure 3:**
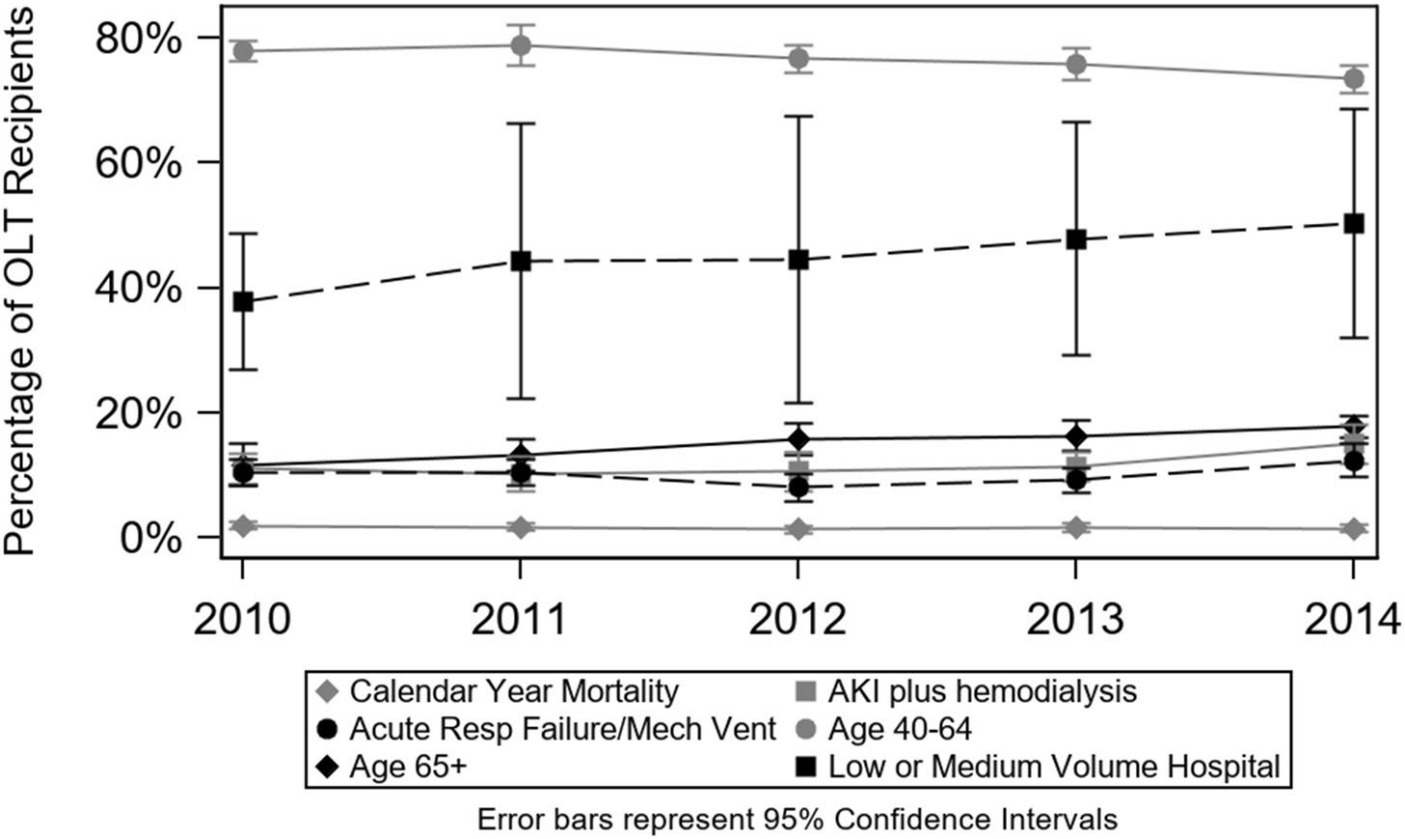
Reasons and procedures performed at early readmissions in liver transplant recipients.

The most common five principal diagnoses for 30-day readmission in all patients were post-operative complications (ICD-9 code—996.82), post-operative infection, electrolyte/fluid abnormalities, gastrointestinal dysfunction and renal impairment. Packed red cell transfusions, biliary interventions, abdominal paracentesis, liver biopsy and GI endoscopy were the most common five interventions during hospital readmission. (Fig. 3a,b). We also performed a subgroup analyses to identify the causes and procedures at readmission in patients > 65 years age. Post-operative complications were lesser in age > 65 (30.1%) as compared to all patients (40.5%). Contrary to that, patients age > 65 years had more transfusions (28.4%) as compared to all patients (22.5%).

### Independent predictors of 30-day readmission in Liver Transplant Recipients

On multivariable logistic regression analysis, Medicaid and Medicare insurance, low and medium volume liver transplant centers, hemodialysis, liver biopsy, infection during the index admission, and LOS greater than 10 days were independent predictors of 30-day readmission after liver transplantation. On the other hand, acute respiratory failure requiring mechanical ventilation was found to be associated with lower odds of readmission within 30 days (adjusted odds ratio 0.84; 95% CI 0.72–0.98) (Table 3). On further analysis, we found that 726/5645 (12.9%) patients who required prolonged mechanical ventilation died during index admission compared to 705/20,832 (3.4%) of patients who did not require prolonged mechanical ventilation (*p* < 0.001). The c-index for the multivariable model was 0.58 (95% confidence interval 0.57–0.60). Patients with LOS greater than 10 days had more comorbidities than patients with LOS ≤ 10 days (mean Elixhauser index 4.7 vs. 4.1, *p* < 0.001).

**Table 3:** Multivariable logistic regression analysis showing predictors of 30-day readmission in liver transplant recipients.

Variable	Multivariable Odds Ratio (95% CI, *p* value)
Primary payer (ref: private insurance)	0.02*
Medicare	1.21 (1.05–1.39, *p* = 0.01)
Medicaid	1.28 (1.05–1.56, *p* = 0.01)
Other/Unknown	0.95 (0.72–1.26, *p* = 0.71)
Hospital Annual Liver Transplant Volume [ref: High (78+)]	< 0.001*
Low (1–48)	1.27 (1.12–1.44, *p* < 0.001)
Medium (49–77)	1.21 (1.03–1.42, *p* = 0.02)
Acute Respiratory Failure w/Mechanical Ventilation	0.84 (0.72–0.98, *p* = 0.03)
Acute Kidney Injury w/Hemodialysis	1.24 (1.05–1.47, *p* = 0.01)
Infection, not including sepsis	1.15 (1.05–1.26, *p* = 0.002)
Liver Biopsy	1.15 (1.02–1.30, *p* = 0.02)
Index admission LOS > 10 days	1.30 (1.16–1.45, *p* < 0.001)

*Omnibus test for overall variable effect.

### Total hospitalization costs of 30-day and calendar year readmissions

The total hospitalization costs of the index hospitalization was greater among patients readmitted within 30 days (Median: $98,067; IQR 71,531–143,466) compared with those who were not readmitted (Median: $87,980; IQR 66,159–134,758), *p* < 0.001 (Table 4). Among patients who were readmitted within 30 days, the median total hospitalization costs of the first readmission was $9960 (IQR 5352–21,242) and the median cumulative total hospitalization costs of all readmissions within 30 days was $12,714 (IQR 6412–28,518). The median total hospitalization costs for the calendar year (index plus all readmissions) was $132,119 (IQR 92,255–209,939) among patients who were readmitted within 30 days compared with a median of $93,577 (IQR 68,635–146,823) among those who were not readmitted within 30 days (Table 4). Most intriguing was the impact of 30 days readmission cost (9.6%; $12,714/$132,119) on the calendar year cost.

**Table 4:** Total Hospital Costs^a^ ($) by 30-day readmission status, median (IQR).

Cost time period	Readmission within 30 days of index discharge (n = 7675; 30.6%)	No Readmission within 30 days of index discharge (n = 17,379; 69.4%)	Effect size	*p* value
Index admission	98,067 (71,531–143,466)^b^	87,980 (66,159–134,758)^b^	0.10	< 0.001
First readmission within 30 days	9960 (5352–21,242)	0	n/a	n/a
All readmissions within 30 days	12,714 (6412–28,518)	0	n/a	n/a
Total calendar year (Index and all readmissions)	132,119 (92,255–209,939)^b^	93,577 (68,635–146,823)^b^	0.35	< 0.001

^a^Adjusted for inflation based on Personal Health Care (Hospital care) Price Indices from the Medical Expenditure Panel Survey (MEPS); costs adjusted to 2014 basis.

^b^n = 7552 non-missing (98.4%).

### 30-day readmission and calendar year mortality

**Figure 4:**
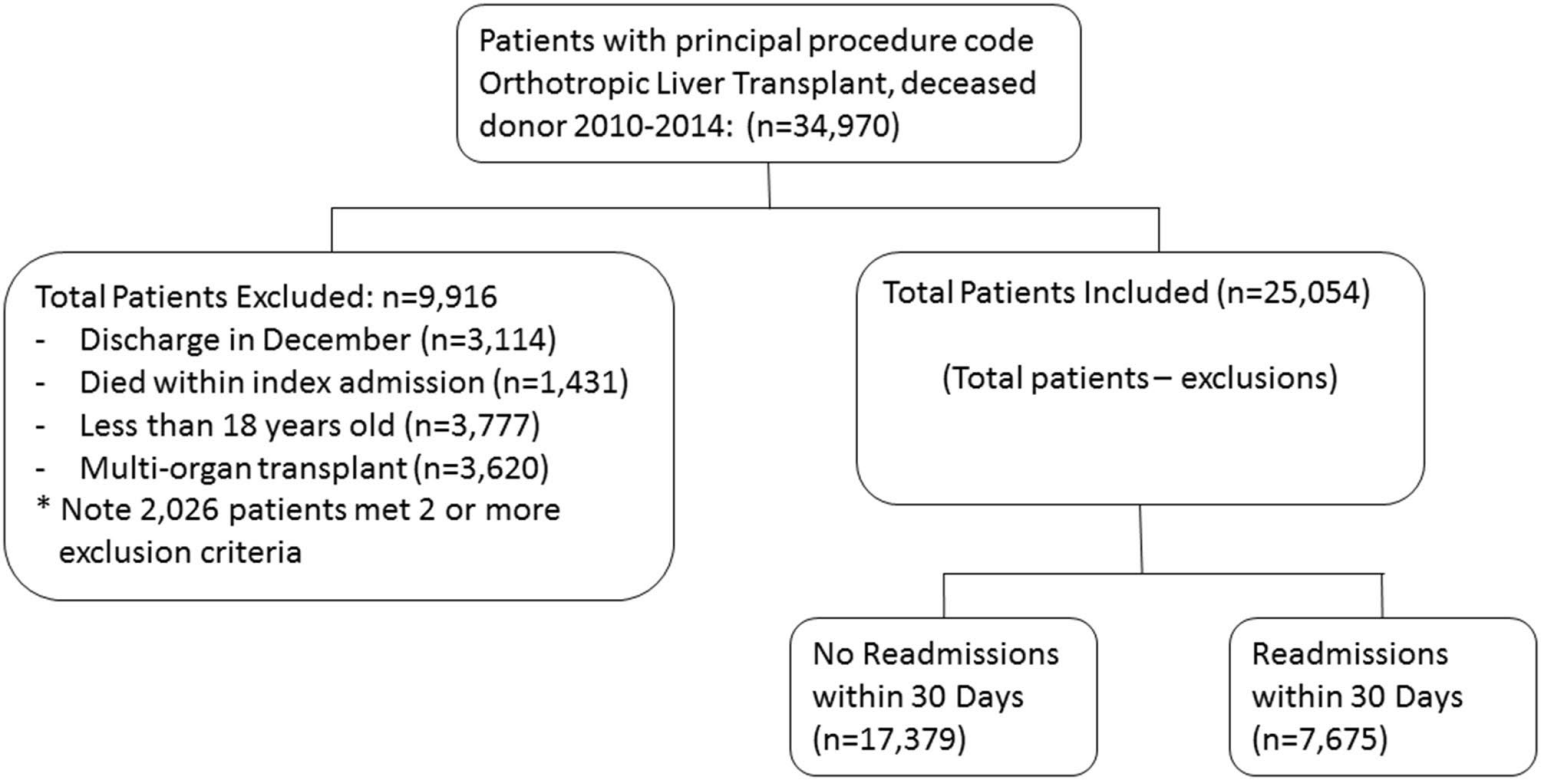
In-hospital mortality including all patients but censoring any mortality within 30 days. Shaded area represent the 95% confidence intervals.

In-hospital mortality was higher among patients readmitted within 30 days (1.8% versus 0.8%, adjusted hazard ratio (aHR): 2.19; 95% CI 1.52–3.17, Fig. 4). Other independent predictors of calendar year mortality were age older than 64 years (aHR 7.89; 95% CI 1.83–34.05) and index admission length of stay greater than 10 days (aHR 1.90; 95% CI 1.05–3.43). Interestingly, alcoholic cirrhosis was a protective factor in this setting (aHR 0.63; CI 0.40–0.98) (Supplemental Table 2). Harrell’s concordance statistic for this model was 0.77 (95% confidence interval 0.73–0.80). In a secondary analyses based on number of readmissions within 30 days, in-hospital mortality did not differ significantly between patients with more than 1 readmission and patients with only one readmission (aHR 1.27; 95% CI 0.59, 2.73).

## Discussion

Using the largest national readmission database in the United States, we show that the 30-day readmission rate after liver transplantation surgery was 30.6% based on 25,054 patients. Between 2010 and 2014, the number of liver transplantation surgeries has increased among patients older than 65 years of age and decreased among those between 40 and 64 years old. Over the same time period, both the inflation adjusted total index hospitalization and calendar year hospitalization costs have also increased significantly. The majority of 30-day readmissions were due to post transplant complications, with packed red blood cell transfusions being the most common intervention during readmission. Independent predictors of 30-day readmission were type of insurance, low and medium volume centers, hemodialysis, liver biopsy, infection, and prolonged LOS. Independent predictors of calendar year in-hospital mortality were 30-day readmission, age older than 64 years, non-alcoholic cirrhosis, and prolonged LOS.

30-day readmission is an important marker of quality of service provided during the index admission. Reduction of hospital readmissions is a declared goal in the United States economic and quality improvement agenda. Previous single center studies reported 30-day readmission rates after liver transplantation of 45%^3,30^. Wilson et al., in a study based on UNOS database, reported a 30-day readmission rate of 38% in this setting. Our analysis revealed a stable trend of annual 30-day readmission rate over the study period of 32–29%, which is lower than that reported in those earlier studies. This improving trend in 30-day readmission rate could be due to the adoption of preventative strategies over time. Russo et al., in a single center study, has reported a reduction in 30-day readmission rate after liver transplantation from 40 to 20% after the implementation of a multi-faceted readmission prevention strategy^4^. The strategy included (1) using observation status instead of inpatient admission when patients sought medical attention after LT and (2) performing same-day or in-office endoscopic retrograde cholangio-pancreatography (ERCP). The telemedicine based readmission prevention protocol proposed by the University of Pennsylvania has significantly decreased the post LT 30-day readmission rates from 32 to 16%^17^. While these and similar interventions can serve as a blue print for individual institutions to build their readmission prevention strategy after, their national reach and generalizability are yet to be determined.

We also report an increasing number of LT among older patients (65 years of age or older), along with an increase in index admission and calendar-year total hospitalization costs and requirement for hemodialysis. Although our study was not designed to identify the reason behind these findings, a possible hypothesis is that the transplant recipients being older and having more comorbidities as is reflected by the increasing requirement for hemodialysis derive the increasing hospitalization costs^18^. In addition, as opposed to the child Pugh Score, creatinine is a component of the MELD score. Therefore, after switching to the MELD score as basis of liver allograft allocation, a larger proportion of patients on the transplant waiting list had renal failure and needed hemodialysis^19^. This increase in the pool of patients with renal failure eligible for liver transplantation might also partially explain the increased rate of hemodialysis and costs post LT.

Our results show that patients in the age groups 18–39 and 40–64 experienced higher 30-day readmission rates compared with patients older than 65 years. On the other hand, Patel et al. found that younger age was associated with lower 90-day readmission rates^3^ after LT. The difference in the results obtained from the two studies can be explained by differences in study designs. Specifically, the study by Patel et al. was a single center study that included 325 patients who received LT between 2005 and 2015. Our study included 25,054 patients from around 2000 hospitals across the United States. While the exact reason for the decreased 30-day readmission rates among older patients is beyond the scope of the current analysis, several studies indicate that a less active immune system might actually benefit older patients receiving LT due to decreased risk of acute rejection^20^. Other possible causes might be poorer compliance with medical instructions and possibly poorer social support system among younger patients.

The association between prolonged LOS and early readmissions after LT we report is consistent with previous literature. Pereira et al. found that index LOS shorter than 9 days was associated with a lower 30-day readmission rate, LOS between 9 and 17 days was associated with higher 30-day readmission rate until the cut-off point of 17 days, after which 30-day readmission rates became lower again^2^. The authors concluded that patients with optimal health likely made up the bulk of the early discharges (LOS less than 9 days) and patients who needed medical optimization may account for the LOS longer than 17 days^2^. In our study more than half of the patients had a post-LT LOS of 11 days. Therefore, based on our and previous studies’ results, we propose the implementation of protocols focusing on reducing LOS during index admission as a measure to decrease 30-day readmission (4). However, the causal relationship between LOS and readmission rate can be complex, since prolonged LOS can lead to debility and thus increase readmission rate. It can also be a marker of more severe disease/higher comorbidity burden which in its turn is the true cause of higher readmission rate.

The median number of days from index discharge to first readmission was 8.1 days (IQR 3.6–15.6). Therefore, we speculate that some of the reasons for readmission could have been identified at the time of discharge or addressed as outpatient.

Acute renal failure, infection, need for a liver biopsy and discharge disposition are potentially modifiable predictors of early readmissions. Infection has been found to be a predictor of readmission in multiple studies^7,21^. Infections after LT is attributable to multiple factors including affection of immunogenic organ (liver), receiving an organ with variable cold ischemia time, being in the hospital, immunosuppressive medications, etc.^22^. All these factors put LTRs to risk of multiple drug resistant bacterial infections (particularly pneumonias, wound infections, cholangitis and bacteremia), as well as viral reactivation (HSV and CMV) and fungemia^23–25^. Liver biopsy is usually performed in the work up of abnormal liver function tests. Those can be due to acute cellular rejection, vascular thromboses, ischemic graft dysfunction or delayed graft function. Therefore, liver biopsy is a marker for these conditions, and implementing protocols to prevent these conditions rather than avoiding liver biopsy itself can potentially help in decreasing 30-day readmission. Approximately 15% of patients were discharged to various outpatient facilities such as skilled nursing facility (SNF), intermediate care facility (ICF) or another type of medical facility. We found that discharge disposition of transfer is an independent predictor of 30-day readmission. Contrary to our finding, Kothari et al. in a study based on 3072 LTRs reported that discharge to skilled nursing facility and inpatient rehabilitation are protective against 30-day readmissions when compared to discharge home or with home health^26^. However, Wilson et al. in a larger study found that disposition to these facilities was indeed a predictor of 30-day readmission^6^. In our study based on NRD, it is assumed that majority of patients return to the same hospital for admission in the immediate post-transplant period, even those living out of state.

Our study is the first to link the type of insurance provider with risk of 30-day readmission. We found Medicare and Medicaid insurance to be independent predictors of 30-day readmission after LT. This finding adds to the growing body of data that establish an association between insurance and disparity in treatment outcomes of multiple medical and surgical conditions. Medicaid insurance and no insurance have been shown to be associated with worse outcomes in the treatment of non-variceal upper gastrointestinal hemorrhage and LT^27,28^. In addition, Medicare/Medicaid primary payer status was also shown to be a 30-day readmission predictor in the post-coronary artery bypass graft population^29^. Along the same line, a national study by Nguyen et al., reviewing the NRD for 2013, revealed Medicaid and Medicare primary care payers as independent predictors of high annual burden and costs of hospitalization for patients with chronic gastrointestinal and liver diseases that are high utilizers of the healthcare system^30^.

There are several studies addressing the association of early readmission with annual hospital LT volume. The majority of those studies have shown better surgical outcomes in liver transplant centers with higher procedure volume^13,14,31,32^. In addition high procedure volume was associated with lower hospital resource utilization. The results of these studies are consistent with those of our national analysis. They suggest that limiting complex surgeries like liver transplantations to centers of excellence with medium to high yearly procedure volume may decrease costs and healthcare resource utilizations.

Our study has certain limitations based on nature of administrative database for research. First, this database relies on ICD-9-CM diagnostic coding and hence is predisposed to inaccurate entries or missing data^33^. However, the rate of missing data among the variables we used was less than 2.0%. In addition, ICD-9-CM codes have been shown to have a high specificity and sensitivity when used to study gastrointestinal diseases^34^. Second, factors such as medication use including immunosuppressants, objective laboratory values and radiology test results are not included in NRD. Donor related information is also not part of NRD. Therefore, polypharmacy, non-compliance with medications and specific immunosuppressive regimens could not be included in our analysis. Further studies with databases that include these factors are needed to clarify their specific contribution to 30-day readmission after LT. Third, for the same reason of limited data variables, we were not able to assess the severity of liver diseases by using the MELD score and the Child–Turcotte-Pugh (CTP) score, which are known predictor of mortality in patients with cirrhosis. However, we used instead well-validated and widely accepted cirrhosis severity classification criteria: the Baveno criteria. The Baveno criteria have been previously used for the stratification of liver cirrhosis severity using administrative databases^35,36^. Fourth, since NRD captures only in-hospital mortality, the 30-day and calendar-year mortality we report might be an underestimate of the true mortality rate. This is because patients who died at home, en-route to the hospital or in the emergency department were not included in our analysis. Lastly, inherent to the NRD’s dependence on state-based data (SID), readmissions occurring in a hospital located in another state could not be captured. However, it is assumed that majority of patients return to the same hospital for admission in the immediate post-transplant period, even those living out of state.

Despite these limitations, our study has several strengths. This study is the most recent, to our knowledge, reporting the 30-day all-cause readmission rate post liver transplantation, its predictors and its impact on treatment outcomes at the national level in the United States. The largest publicly available all-payer readmission database in the United States is used that minimized the likelihood of a beta error. Most importantly, the NRD is nationally representative and includes patients from hospitals that are small, medium, and large; rural & urban; privately or publicly owned; teaching & non-teaching; and for profit & not for profit, across 18–22 states. This makes the study results more readily generalizable to the United States. Furthermore, the unique variables in the database permitted us to explore factors such as hospitalization costs, household income estimates, insurance carrier and hospital factors, which are not commonly available in single-center and UNOS database studies.

In conclusion, this is the first study based on nationwide readmission database to determine the 30-day all cause readmission rate after liver transplantation, as well as its risk factors and impact on patient’s outcome. We found that the 30-day readmission rate is 30.6%. Between 2010 and 2014, the number of liver transplantation surgeries has increased among patients older than 65 years of age and decreased among those between 40 and 64 years old. Over the same time period, both the inflation adjusted total index hospitalization and calendar year hospitalization costs have also increased significantly. The majority of 30-day readmissions were due to post transplant complications. Independent predictors of 30-day readmission were type of insurance, low & medium volume centers, hemodialysis, liver biopsy, infection, and prolonged LOS. Independent predictors of calendar year in-hospital mortality were 30-day readmission, age older than 64 years, and prolonged LOS.

Early readmission not only increases economic burden on healthcare, but is also associated with increased calendar year mortality. The data we present on early readmission, its predictors, trends of liver transplantation and healthcare utilization can be helpful to the patients, clinicians, payers, and transplantation policymakers. Addressing potentially modifiable predictors of readmission, such as type of insurance, center volume, requirement of HD and discharge disposition may reduce the readmission rate. Although other predictors may not be modifiable, they can be used to identify patients at high risk of readmission, and who would thus benefit the most from interventions aimed at decreasing 30-day readmissions. Future interventions for all patients, and especially those at high risk for readmission, have the potential to decrease readmission rates and associated health care expenditures, as well as improve morbidity and mortality from liver transplantation.

## Supplementary information

Supplementary table 1.

Supplementary table 2.
