# Supplementary material for: Thirty-day readmission rates, trends and its impact on liver transplantation recipients: a national analysis
Source: Sci Rep. 2020 Nov 6;10:19254. doi: 10.1038/s41598-020-76396-5 (PMC7648628; doi:10.1038/s41598-020-76396-5)
Supplement: Supplementary file 1 — Supplementary table 1. [file 41598_2020_76396_MOESM1_ESM.docx]

**Thirty-Day Readmission Rates, Trends and its Impact on Liver Transplantation Recipients: A National Analysis**

Khalid Mumtaz MBBS, MSc, Jannel Lee-Allen, Kyle Porter MAS, Sean Kelly MD, James Hanje MD, Lanla F. Conteh MD, MS, Anthony J. Michaels MD, Ashraf El-Hinnawi MD, Ken Washburn MD, Sylvester M. Black MD, PhD, Marwan S. Abougergi, MD.

**Supplementary table** 1. ICD-9-CM codes used for data extraction and analysis from the Nationwide Readmission Database 2013

| **Diagnosis or Procedure** | **ICD-9-CM Codes Used** | **Variable location** |
| --- | --- | --- |
| Cirrhosis – Non – Alcoholic | 571.5, 571.6 and not 303.XX (alcoholic dependence syndrome) | DX1-DX30 |
| Cirrhosis – Alcoholic | 571.2 OR  571.X with 303.XX (alcoholic dependence syndrome) | DX1-DX30 |
| Decompensated Cirrhosis | Any cirrhosis code (571.2, 571.5, 571.6) AND at least one of Ascites, Encephalopathy, Variceal Bleeding, Hepatorenal Syndrome, or Portal Hypertension (below) |  |
| Ascites | 789.5, 789.59 | DX1-DX30 |
| Encephalopathy | 348.30, 348.39, 572.2, 780.97 | DX1-DX30 |
| Variceal bleeding | 456.0 | DX1-DX30 |
| Hepatorenal syndrome | 572.4 | DX1-DX30 |
| Portal hypertension | 572.3 | DX1-DX30 |
| Spontaneous bacterial peritonitis | 567.23 | DX1-DX30 |
| HCC | 155.0 | DX1-DX30 |
| Diabetes | 250.XX | DX1-DX30 |
| **Other organ transplants** |  |  |
| Kidney | 55.6X | DX1-DX30 |
| Heart | 37.5X | DX1-DX30 |
| Lung | 33.5X | DX1-DX30 |
| Pancreas | 52.8X | DX1-DX30 |
| **Post LT complications** |  |  |
| Acute Respiratory failure and  Mechanical ventilation | 518.0, 518.81, 518.82, 518.84  93.90, 96.01, 96.02, 96.03, 96.04, 96.05, 96.70, 96.71, 96.72 | DX1-DX30  PR1-PR15 |
| Acute kidney injury and  Hemodialysis | 584.5, 584.6, 584.7, 584.8, 584.9, 586  39.95 | DX1-DX30  PR1-PR15 |
| Intra-abdominal infections | 567.2, 567.1, 567.29, 567.22, 567.38, 567.39 | DX1-DX30 |
| Sepsis, Septic Shock | 785.52, 038, 995.9, 020.2, 790.7, 117.9, 112.5 | DX1-DX30 |
| Post-Operative Shock | 998.0 | DX1-DX30 |
| Hepatic artery thrombosis | 444.89 | DX1-DX30 |
| Infectious | 576.1, 785, 572.0, 998.59, 780.60, 038.9, 486, 599.0, 008.45, 682.6, 790.7, 465.9, 038.42, 038.49, 998.02, 998.59, 038.0, 995.91,999.31,567.22, 078.5,075, 567.22, 998.59 | DX1-DX30 |
| CDI | 008.45 | DX1-DX30 |

**Biliary complications:**

| **Procedure** | **ICD-9 Codes Used** | **Variable Location** |
| --- | --- | --- |
| Bile duct obstruction | 576.2,576.8 | DX1-DX30 |
| ERCP | 51.10, 51.11, 51.14, 52.13, 52.14, 51.84-51.88, 51.95, 51.99 | PR1-PR15 |
| PTDB | 52.93, 52.94, 52.97, 52.98, 51.96, 51.98, 51.43, 51.12, 51.59, 97.55 | PR1-PR15 |
| Bile duct anastomosis repair | 51.39 | PR1-PR15 |
| Bile duct exploration | 51.42 | PR1-PR15 |

**Post LT Procedural Codes:**

| **Procedure** | **ICD-9 Codes Used** | **Variable Location** |
| --- | --- | --- |
| Liver Transplantation [2] | 50.59 (OLT) [4] | PR1-PR15 |
| Deceased Donor | 00.93 | PR1-PR15 |
| Living Donor | 00.91, 00.92 | PR1-PR15 |
| Acute Respiratory failure and mechanical ventilation | 93.90, 96.01, 96.02, 96.03, 96.04, 96.05, 96.70, 96.71, 96.72 | PR1-PR15 |
| Infusion of intravenous vasopressor | 00.17 | PR1-PR15 |
| Liver biopsy | 50.11-50.19, | PR1-PR15 |
| Acute kidney injury and hemodialysis | 39.95 | PR1-PR15 |
| Post-surgery Laparotomy [8] | 39.98, 54.12 (Re-open), 54.1, 54.11, 54.19 | PR1-PR15 |
| Blood Transfusion [6] | 99.00, 99.02, 99.03, 99.04, 99.05, 99.05, 99.07 | PR1-PR15 |
