# Supplementary material for: Thirty-day readmission rates, trends and its impact on liver transplantation recipients: a national analysis
Source: Sci Rep. 2020 Nov 6;10:19254. doi: 10.1038/s41598-020-76396-5 (PMC7648628; doi:10.1038/s41598-020-76396-5)
Supplement: Supplementary file 2 — Supplementary table 2. [file 41598_2020_76396_MOESM2_ESM.docx]

**Thirty-Day Readmission Rates, Trends and its Impact on Liver Transplantation Recipients: A National Analysis**

Khalid Mumtaz MBBS, MSc, Jannel Lee-Allen, Kyle Porter MAS, Sean Kelly MD, James Hanje MD, Lanla F. Conteh MD, MS, Anthony J. Michaels MD, Ashraf El-Hinnawi MD, Ken Washburn MD, Sylvester M. Black MD, PhD, Marwan S. Abougergi, MD.

**Supplemental Table 2. Predictors of calendar year mortality in liver transplant recipients**

| **Variable** | **Multivariable Hazard Ratio**  **(95% CI, p-value)** |
| --- | --- |
| Readmission within 30 days | 2.19 (1.54-3.12, p<0.001) |
| Age Group (ref: 18-39) | p=0.04* |
| 40-64 | 3.24 (0.71-14.68, p=0.13) |
| >64 | 7.89 (1.86-33.50, p=0.01) |
| Index admissions length of stay > 10 days | 1.90 (1.07-3.37, p=0.03) |
| Alcoholic vs non-alcoholic Cirrhosis | 0.63 (0.41-0.97, p=0.03) |

* omnibus test for overall variable effect
